# Development of a nomogram for predicting renal survival in patients with biopsy-proven diabetic nephropathy

**DOI:** 10.3389/fendo.2025.1532494

**Published:** 2025-03-27

**Authors:** Zishan Lin, Tao Hong, Wenfeng Wang, Shidong Xie, Xiaohong Zhang, Xuan Tao, Feng Yang, Caiming Chen, Dewen Jiang, Jianxin Wan, Hong Chen, Yanfang Xu

**Affiliations:** ^1^ Department of Nephrology, Blood Purification Research Center, The First Affiliated Hospital, Fujian Medical University, Fuzhou, China; ^2^ Research Center for Metabolic Chronic Kidney Disease, The First Affiliated Hospital, Fujian Medical University, Fuzhou, China; ^3^ Department of Nephrology, National Regional Medical Center, Binhai Campus of the First Affiliated Hospital, Fujian Medical University, Fuzhou, China; ^4^ Department of Pathology, The First Affiliated Hospital, Fujian Medical University, Fuzhou, China; ^5^ Department of Nephrology, Fuzhou No. 1 Hospital Affiliated with Fujian Medical University, Fuzhou, China

**Keywords:** type 2 diabetes mellitus, diabetic nephropathy, end-stage renal disease, prognostic model, nomogram

## Abstract

**Background:**

Diabetic nephropathy (DN) has emerged as the leading cause of chronic kidney disease, with a significant proportion of DN patients progressing to end-stage kidney disease (ESKD), profoundly affecting their quality of life. Currently, no single clinical marker reliably predicts the likelihood and timing of progression to ESKD in DN patients. This study aims to develop a non-invasive predictive model to evaluate the risk and timing of ESKD onset in this population.

**Methods:**

This study retrospectively analyzed data from 140 biopsy-confirmed DN patients. Key predictive variables were identified using multivariate Cox regression analysis, and a visual predictive nomogram was developed. The model was subsequently evaluated for its predictive performance.

**Results:**

Of the 140 DN patients, 81 progressed to ESKD. Multivariate analysis identified estimated glomerular filtration rate, common logarithm of albumin-creatinine ratio, cystatin C, hemoglobin, and fibrinogen as independent predictors of progression to ESKD. Based on these significant factors, a nomogram was constructed. The area under the time-dependent receiver operating characteristic curve at 1, 2, 3, and 5 years were 0.898 (95% CI: 0.839–0.958), 0.889 (95% CI: 0.818–0.959), 0.876 (95% CI: 0.785–0.968), and 0.893 (95% CI: 0.796–0.990), respectively. Calibration curves demonstrated strong concordance between predicted and observed outcomes, while decision curve analysis indicated substantial net clinical benefit for practical application.

**Conclusions:**

This study developed a predictive model to assess the risk and timing of ESKD progression in DN patients. As a quantitative tool, this model enables clinicians to estimate the 5-year risk of ESKD, facilitating timely interventions to improve patient outcomes.

## 1 Introduction

Diabetic nephropathy (DN) is one of the most serious microvascular complications of diabetes, affecting approximately 20%-40% of diabetic patients (1). As the prevalence of diabetes increases, the incidence of DN rises correspondingly, placing substantial burdens on both individuals and healthcare systems (2, 3). DN is a primary cause of chronic kidney disease (CKD) and end-stage kidney disease (ESKD) worldwide. In 2019, the Centers for Disease Control and Prevention highlighted that nearly half of the new ESKD cases in the United States were due to diabetes (4). Moreover, DN notably increases mortality among type 2 diabetes patients (5). Early detection of DN patients at risk of progressing to ESKD is vital to mitigate its severe outcomes, including quality of life deterioration, increased mortality, and escalated healthcare costs (4, 6).

Current tools for predicting DN progression are inadequate. While albuminuria and estimated glomerular filtration rate (eGFR) are commonly employed, their predictive accuracy is sometimes limited (7). For instance, non-albuminuric DN can progress to CKD regardless of initial eGFR levels, and decreased eGFR shows a strong correlation with adverse outcomes primarily in patients with significant proteinuria, with less clarity in early-stage disease (8, 9). These challenges underscore the necessity for more reliable predictive markers. Recent studies suggested that certain pathological features, such as interstitial fibrosis and tubular atrophy (IFTA), can be used to assess the prognosis of DN (7, 10). However, not all DN patients undergo renal biopsy, and such assessments require experienced renal pathologists.

Given these challenges, there is a critical need to develop a robust, user-friendly risk assessment tool that assists clinicians in quickly identifying high-risk DN patients and accurately estimating their progression risk. Such a tool would facilitate more effective and targeted monitoring strategies, potentially improving the management of factors associated with DN progression. In this study, we aim to derive a new predictive model using routine laboratory parameters to assess the risk of ESKD in patients with biopsy-confirmed DN. The model is designed to be both practical and cost-effective, enabling its broader adoption in healthcare settings.

## 2 Methods

### 2.1 Study design and patient selection

This retrospective study analyzed patients with Type 2 diabetes mellitus (T2DM) who were diagnosed with DN through percutaneous renal biopsy at the First Affiliated Hospital of Fujian Medical University and Fuzhou NO.1 Hospital Affiliated with Fujian Medical University between January 1, 2014, and May 31, 2023. The primary endpoint was progression to ESKD, defined as an eGFR <15 mL/min/1.73 m², initiation of long-term dialysis, or kidney transplantation. The inclusion criteria were: age >14 years, a diagnosis of T2DM, and confirmation of DN by renal biopsy. Exclusion criteria included patients with a baseline eGFR of less than 15 mL/min/1.73 m², those with renal biopsy findings suggesting DN combined with other renal diseases, patients with incomplete clinical data or unclear follow-up status, and patients with other organ failures or active infections.

### 2.2 Data collection

This study collected baseline clinical data for all patients prior to renal biopsy, including demographic information, medical history, laboratory and imaging tests, and renal biopsy pathology reports. Demographic and medical history data included age, sex, BMI, and history of conditions such as diabetes, diabetic retinopathy, and hypertension. Laboratory and imaging test data consisted of white blood cell count (WBC), hemoglobin (Hb), C-reactive protein (CRP), glycated hemoglobin (HbA1c), serum creatinine (SCr), blood urea nitrogen (BUN), uric acid (UA), eGFR, albumin (Alb), cystatin C (CysC), fibrinogen (Fib), urinary albumin-creatinine ratio (ACR), and various other parameters obtained from urine and imaging tests. Blood samples were collected after overnight fasting for more than 8 hours, and all samples were analyzed using commercially available test kits and automated chemical analyzers.

The criteria for renal biopsy in this study were as follows: proteinuria >3.5 g/day with normal renal function; presence of glomerular hematuria; rapid decline in renal function within 3 months; presence of renal dysfunction without diabetic retinopathy; and degree of proteinuria and renal dysfunction not consistent with the duration of diabetes (e.g., diabetes duration <5 years). Kidney biopsy specimens were obtained through ultrasound-guided procedures, formalin-fixed, paraffin-embedded, and sectioned to a thickness of 2 μm. Staining methods including Hematoxylin and Eosin (HE), Periodic Acid-Silver Methenamine (PASM), Masson trichrome, and Congo Red were employed, along with immunofluorescence using specific antibodies. Fresh frozen tissue samples were used for the immunofluorescence staining. Kidney biopsy samples were also analyzed using electron microscopy. Pathological results were reviewed by two experienced nephropathologists, and in cases of disagreement, a third pathologist was consulted.

### 2.3 Definitions

Diabetic retinopathy was diagnosed by an ophthalmologist based on diabetes history and characteristic changes seen in fundoscopic exams. Hypertension was defined as systolic blood pressure ≥140 mmHg and/or diastolic blood pressure ≥90 mmHg based on three non-consecutive measurements. eGFR was calculated using the Chronic Kidney Disease Epidemiology Collaboration (CKD-EPI) equation, and kidney volume was estimated using the ellipsoid formula: volume (cm³) = length (cm) × width (cm) × height (cm) × 0.523 (11). The common logarithm of ACR (lgACR) was calculated using base-10 logarithms of the ACR values. For grouping purposes, eGFR < 60 mL/min/1.73m² was chosen based on the KDIGO guidelines as a marker of CKD. Hb was categorized using the World Health Organization anemia classification, with Hb < 110 g/L serving as the cutoff for mild anemia in both males and females. As there are no universally accepted cutoff values for lgACR, CysC, and Fib, these parameters were grouped based on the median values of the cohort.

### 2.4 Statistical analyses

All statistical analyses were conducted using SPSS 26.0 (SPSS Inc., Chicago, IL, USA) and R studio 4.4.0. Graphs were optimized using GraphPad Prism 9.5.0. Categorical variables were expressed as numbers and percentages, while continuous variables were presented as mean ± standard deviation (SD) if normally distributed, or as medians with interquartile ranges (IQR) for non-normal distributions.

Comparisons between groups were performed using the t-test for continuous variables with normal distributions, the Kruskal-Wallis test for non-normally distributed continuous variables, and the chi-square test for categorical variables. Correlations between variables were evaluated using Spearman’s rank correlation coefficient. Renal survival between groups was compared using Kaplan-Meier curves.

Initially, univariate Cox regression analysis was performed to assess the impact of various factors on renal survival in DN patients. Independent risk factors were identified through multivariate Cox regression analysis (stepwise forward LR), and survival curves were used to validate these findings, ultimately constructing the nomogram. Time-dependent receiver operating characteristic curves (td-ROC) were used to assess the discriminatory ability of the nomogram at different time points. Calibration curves were plotted to compare observed and predicted probabilities. The accuracy of the best-performing model was assessed using the Brier score, a quantitative measure of calibration. It is defined as the mean squared difference between the observed and predicted outcomes, with values ranging from 0 to 100%, where 0 represents the best possible calibration. A lower Brier score indicates better accuracy in predicting probabilities and reflects a more reliable model. Decision curve analysis (DCA) was used to evaluate the clinical utility of the model by estimating the net benefit for clinical decision-making. A two-sided P-value < 0.05 was considered statistically significant

## 3 Results

### 3.1 Patient characteristics

A total of 140 patients were included in this study. Baseline characteristics of the patients are shown in **Table 1** and **Supplementary Table 1**. The median age of the cohort was 54 years (IQR 46–60), with 99 male (70.7%) and 41 female (29.3%). The median duration of diabetes was 96 months (IQR 60–144), and the median duration of kidney disease was 6 months (IQR 3–24). A total of 121 patients (86.4%) had diabetic retinopathy, and 123 (87.9%) had hypertension. The mean BMI was 23.75 ± 2.87 kg/m². Regarding laboratory results, the median eGFR, CysC, and Fib levels were 48.78 mL/min/1.73 m² (IQR 34.83–75.46), 1.53 mg/L (IQR 1.17–1.92), and 4.53 g/L (IQR 3.71–5.77), respectively, with 90 patients (64.3%) having an eGFR <60 mL/min/1.73 m². The mean Hb level was 107.41 ± 22.76 g/L, and the median urinary total protein (UTP) level was 4.24 g/24h (IQR 2.76–7.52).

**Table 1:** Demographic and clinical data of patients with diabetes nephropathy.

Characteristics	Total (*n*=140)	Progression to ESKD		P
		No (*n*=59)	Yes (*n*=81)	
Male, *n* (%)	99 (70.7%)	47 (79.7%)	52 (64.2%)	0.047
Age (years), median (IQR)	54 (46~60)	56 (46~64)	52 (46~59)	0.046
Duration of follow up (months)	12 (4~24)	15 (2~35)	10 (6~19)	0.226
Duration of diabetes (months)	96 (60~144)	96 (60~120)	96 (48~156)	0.847
Duration of kidney disease (months)	6 (3~24)	12 (3.96~24)	6 (3~12)	0.349
Diabetic retinopathy, *n* (%)	121 (86.4%)	45 (76.3%)	76 (93.8%)	0.003
Hypertension, *n* (%)	123 (87.9%)	53 (89.8%)	70 (86.4%)	0.542
BMI (kg/m^2^), mean ± SD	23.75 ± 2.87	23.89 ± 2.98	23.64 ± 2.80	0.601
eGFR (mL/min/1.73 m^2^)	48.78 (34.83~75.46)	66.88 (49.24~89.74)	40.10 (24.21~51.04)	<0.001
BUN (mmol/L)	9.07 (6.60~13.05)	7.47 (6.37~9.44)	10.93 (7.36~15.57)	<0.001
SCr (μmol/L)	131.10 (96.15~175.53)	108.80 (76.00~134.00)	159.00 (121.55~239.30)	<0.001
Hematuria, *n* (%)	115 (82.1%)	46 (80.0%)	69 (85.2%)	0.271
Urinary cast, (/LPF)	1.62 (0.48~4.86)	1.62 (0.38~4.06)	1.86 (0.74~5.31)	0.206
UTP (g/24h)	4.24 (2.76~7.52)	3.49 (1.69~5.70)	4.9 (3.13~8.99)	0.004
lgACR (mg/g)	3.54 (3.27~3.71)	3.35 (3.08~3.58)	3.62 (3.42~3.78)	<0.001
Kidney volume (cm^3^)	180.18 (152.15~181.38)	180.16 (140.4~181.11)	180.37 (162.27~181.61)	0.063
WBC (× 10^9^/L)	6.78 (5.61~8.08)	6.69 (5.27~7.54)	6.97 (5.68~8.42)	0.157
Hb (g/L)	107.41 ± 22.76	118.66 ± 20.82	99.21 ± 20.59	<0.001
PLT (× 10^9^/L)	242.00 (196.50~305.75)	233.00 (185.00~278.00)	248.00 (212.00~322.50)	0.013
HbA1c (%)	7.75 (6.43~9.10)	7.80 (6.60~9.10)	7.60 (6.40~9.30)	0.572
Uric acid (μmol/L)	380.10 (325.00~433.70)	366.60 (326.35~425.08)	385.00 (319.70~434.15)	0.387
Albumin (g/L)	29.77 ± 6.28	29.80 (25.60~34.90)	28.00 (25.25~33.75)	0.338
CysC (mg/L)	1.53 (1.17~1.92)	1.30 (1.07~1.66)	1.68 (1.31~2.10)	<0.001
Triglycerides (mmol/L)	1.63 (1.11~2.48)	1.57 (1.09~1.98)	1.76 (1.16~2.82)	0.106
Total cholesterol (mmol/L)	5.29 (4.23~6.83)	5.04 (4.10~6.34)	5.33 (4.32~7.11)	0.341
HCO3- (mmol/L)	24.80 (22.5~27.58)	24.92 ± 3.62	24.44 ± 3.85	0.461
CRP (mg/L)	5.00 (3.55~5.99)	5.00 (3.38~8.58)	5.00 (3.55~5.72)	0.986
Fib (g/L)	4.53 (3.71~5.77)	4.15 (3.37~5.55)	4.75 (4.14~6.09)	0.004
D-dimer (mg/L)	0.81 (0.44~1.66)	0.71 (0.39~1.43)	0.91 (0.51~1.74)	0.288

BMI, body mass index; BUN, blood urea nitrogen; UTP, urine total protein quantity; ACR, albumin-to-creatinine ratio; WBC, white blood cell; PLT, platelet; Hb, hemoglobin; CysC, cystatin C; CRP, C-reactive protein; Fib, fibrinogen.

By the end of the follow-up period, 81 patients (57.9%) had progressed to ESKD, while 59 patients (42.1%) had not. Patients were divided into two groups based on their ESKD status. Compared to the non-ESKD group, patients in the ESKD group were younger, had a higher proportion of males, and lower levels of eGFR and Hb, while showing significantly higher levels of BUN, SCr, PLT, CysC, Fib, UTP, and lgACR.

### 3.2 Screening potential predictors of renal survival

The median renal survival time for the cohort was 10 months, with a median follow-up duration of 12 months. Among the 140 patients, 81 (57.9%) reached the follow-up endpoint, with 52 patients (64.2%) reaching the endpoint within one year, 70 patients (86.4%) within two years, and 80 patients (98.8%) within five years. One remaining patient reached the endpoint at the 65th month of follow-up. As listed in **Table 2**, univariate Cox regression analysis identified the following factors as significant risk factors for progression to ESKD in DN patients: age, eGFR, BUN, SCr, diabetic retinopathy, Hb, PLT, CysC, HCO3-, Fib, D-dimer, UTP, lgACR, urinary cast, and kidney volume.

**Table 2:** Univariate Cox regression analysis of factors associated with progression to ESKD in diabetic nephropathy patients.

Variables	Univariate		95% CI	
	HR (95% CI)	P	HR (95% CI)	P
Age (years)	0.978 (0.958, 0.998)	0.029		
eGFR (mL/min/1.73 m^2^)	0.963 (0.952, 0.975)	<0.001	0.979 (0.966, 0.993)	0.004
BUN (mmol/L)	1.024 (1.012, 1.036)	<0.001		
SCr (μmol/L)	1.010 (1.008, 1.012)	<0.001		
Diabetic retinopathy	1.004 (1.101, 6.763)	0.030		
Hb (g/L)	0.969 (0.959, 0.980)	<0.001	0.981 (0.969, 0.993)	0.003
PLT (× 10^9^/L)	1.003 (1.001, 1.005)	0.005		
CysC (mg/L)	2.530 (1.914, 3.344)	<0.001	1.897 (1.197, 3.007)	0.006
HCO3- (mmol/L)	0.911 (0.854, 0.973)	0.005		
Fib (g/L)	1.241 (1.080, 1.427)	0.002	1.219 (1.021, 1.456)	0.028
D-dimer (mg/L)	1.155 (1.006, 1.328)	0.041		
UTP (g/24h)	1.120 (1.058, 1.187)	<0.001		
lgACR (mg/L)	5.635 (2.571, 12.350)	<0.001	2.765 (1.186, 6.446)	0.019
Urinary cast, (/LPF)	1.076 (1.028, 1.127)	0.002		
Kidney volume (cm^3^)	1.010 (1.001, 1.020)	0.039		

### 3.3 Identification of independent predictors of renal survival

Based on the results of the univariate Cox regression, further multivariate Cox regression analysis was performed. The results revealed that eGFR (HR 0.979, 95% CI 0.966–0.993, P = 0.004), lgACR (HR 2.765, 95% CI 1.186–6.446, P = 0.019), CysC (HR 1.897, 95% CI 1.197–3.007, P = 0.006), Hb (HR 0.981, 95% CI 0.969–0.993, P = 0.003), and Fib (HR 1.219, 95% CI 1.021–1.456, P = 0.028) were independent risk factors for progression to ESKD in DN patients (**Table 2**).

**Figure 1:**
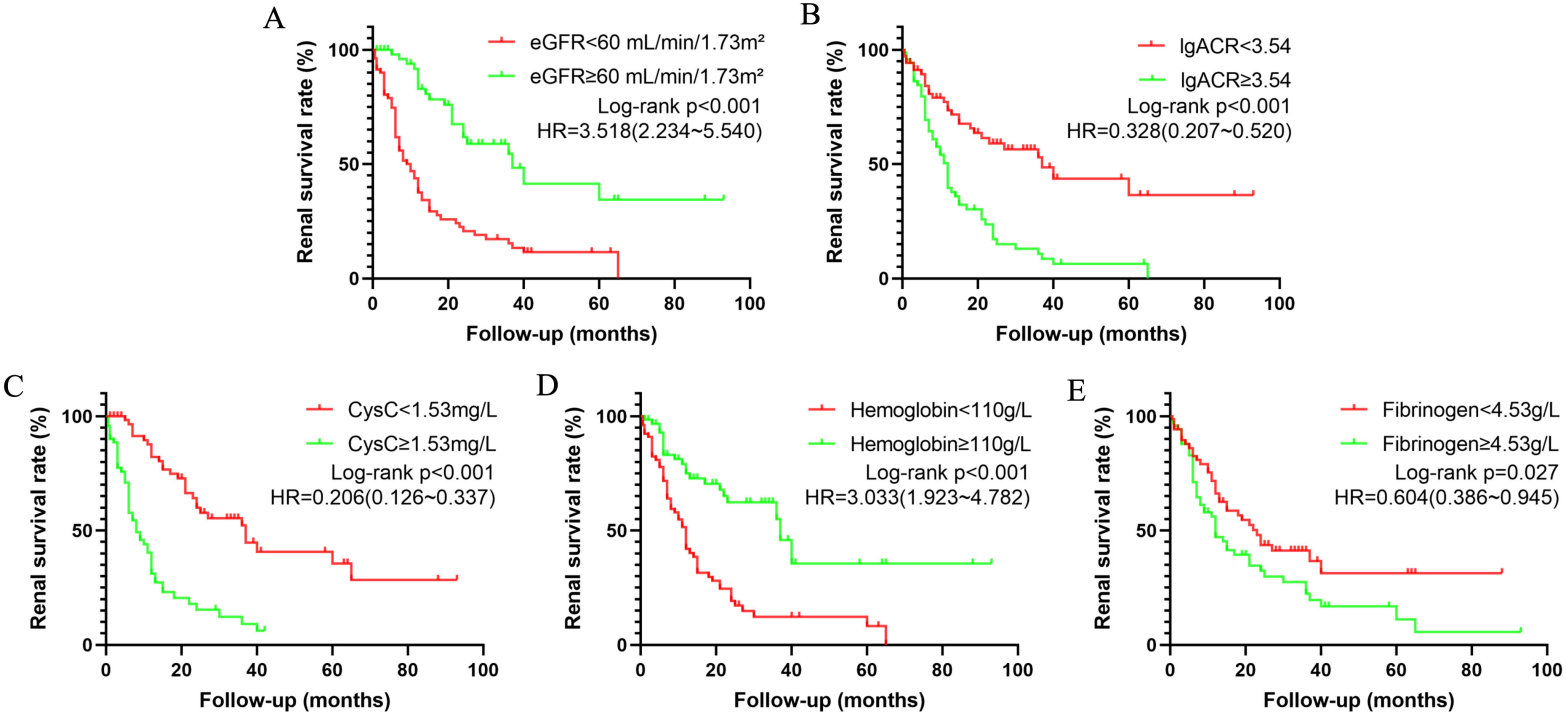
Kaplan-Meier survival curves illustrating renal survival in patients with diabetic nephropathy (DN) based on various risk factors: eGFR **(A)**, lgACR **(B)**, CysC **(C)**, Hb **(D)**, and Fib **(E)**. eGFR refers to estimated glomerular filtration rate; lgACR denotes the common logarithm of the albumin-to-creatinine ratio; CysC stands for Cystatin C.

Based on the identified independent risk factors, DN patients were grouped according to whether their eGFR was <60 mL/min/1.73m², lgACR <3.54 mg/g, CysC <1.53 mg/L, Hb <110 g/L, and Fib <4.53 g/L. Survival curves were plotted to further analyze the impact of each independent risk factor on renal survival in DN patients. As shown in **Figure 1**, eGFR, lgACR, CysC, Hb, and Fib were significant risk factors for renal survival and can thus serve as predictors for progression to ESKD in DN patients, supporting their inclusion in a predictive model.

### 3.4 Development of prognostic nomogram

**Figure 2:**
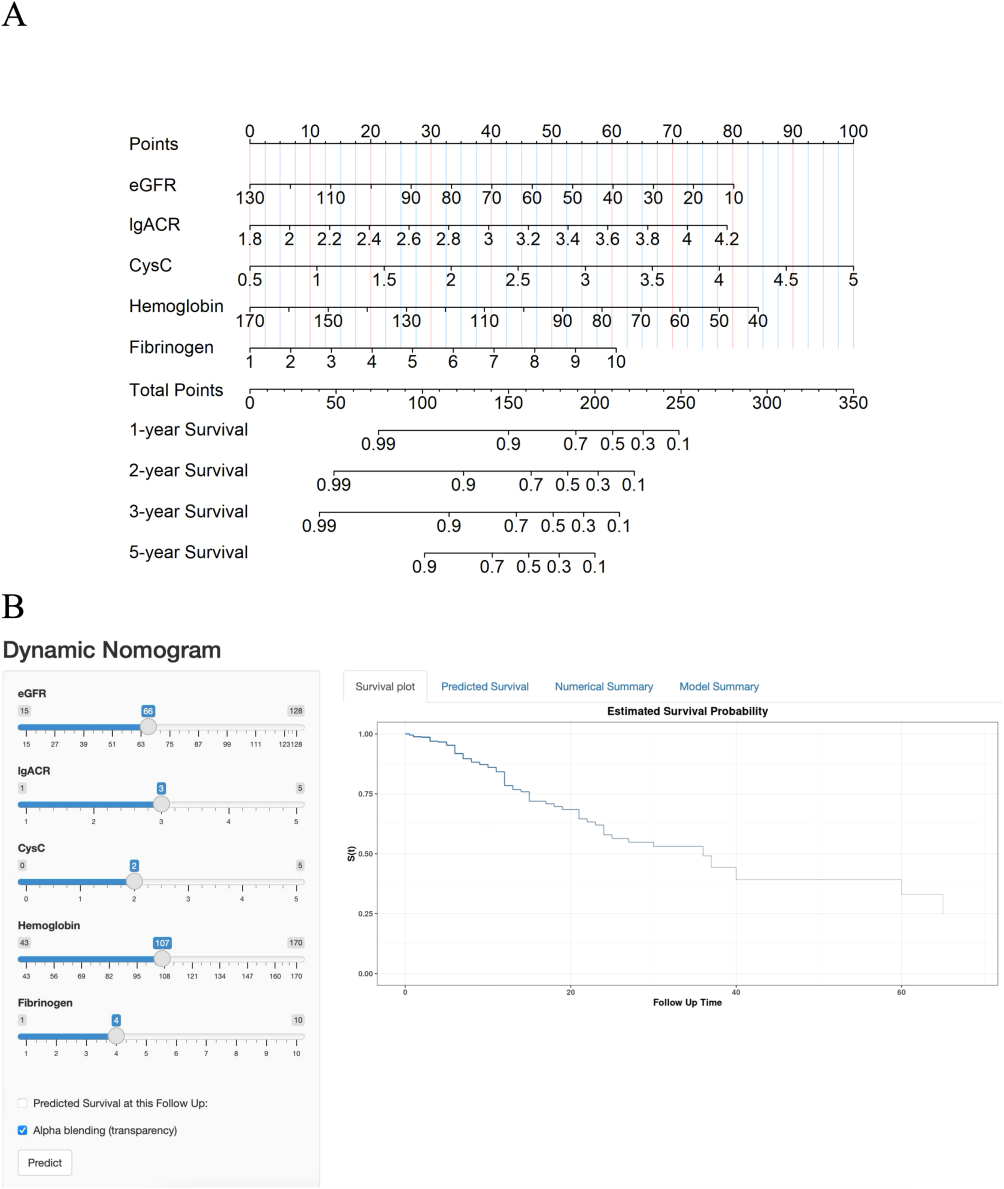
**(A)** Nomogram for predicting the progression of DN patients to end-stage kidney disease (ESKD). **(B)** Online dynamic nomogram.

According to the results from the multivariate Cox regression analysis, we developed a nomogram to predict the risk of ESKD progression in DN patients at 1, 2, 3, and 5 years (**Figure 2A**). The nomogram incorporates five predictive variables: eGFR, lgACR, CysC, Hb, and Fib, each assigned a specific score based on its weight. To use the nomogram, locate the value of each variable on the corresponding axis, draw a straight line upward to the points scale, and sum the scores. The total score is then mapped to the survival probability scale at 1, 2, 3, and 5 years, providing an estimate of the likelihood of progression to ESKD.

To facilitate the use of this model, we also developed an online dynamic nomogram. This interactive tool allows clinicians to input individual patient variables and obtain corresponding survival curves. The online dynamic nomogram can be accessed at the following link: https://hongtao123456.shinyapps.io/DynNomapp/(**Figure 2B**). However, due to the limitation that only integer values can be input, the tool has some restrictions in use. We recommend using the online dynamic nomogram for a rough estimation of a patient’s survival curve, followed by using the static nomogram for a more detailed calculation of risk probabilities.

These methods enable clinicians to assess the risk of ESKD progression over various time points, facilitating timely interventions.

### 3.5 Assessment of nomogram

**Figure 3:**
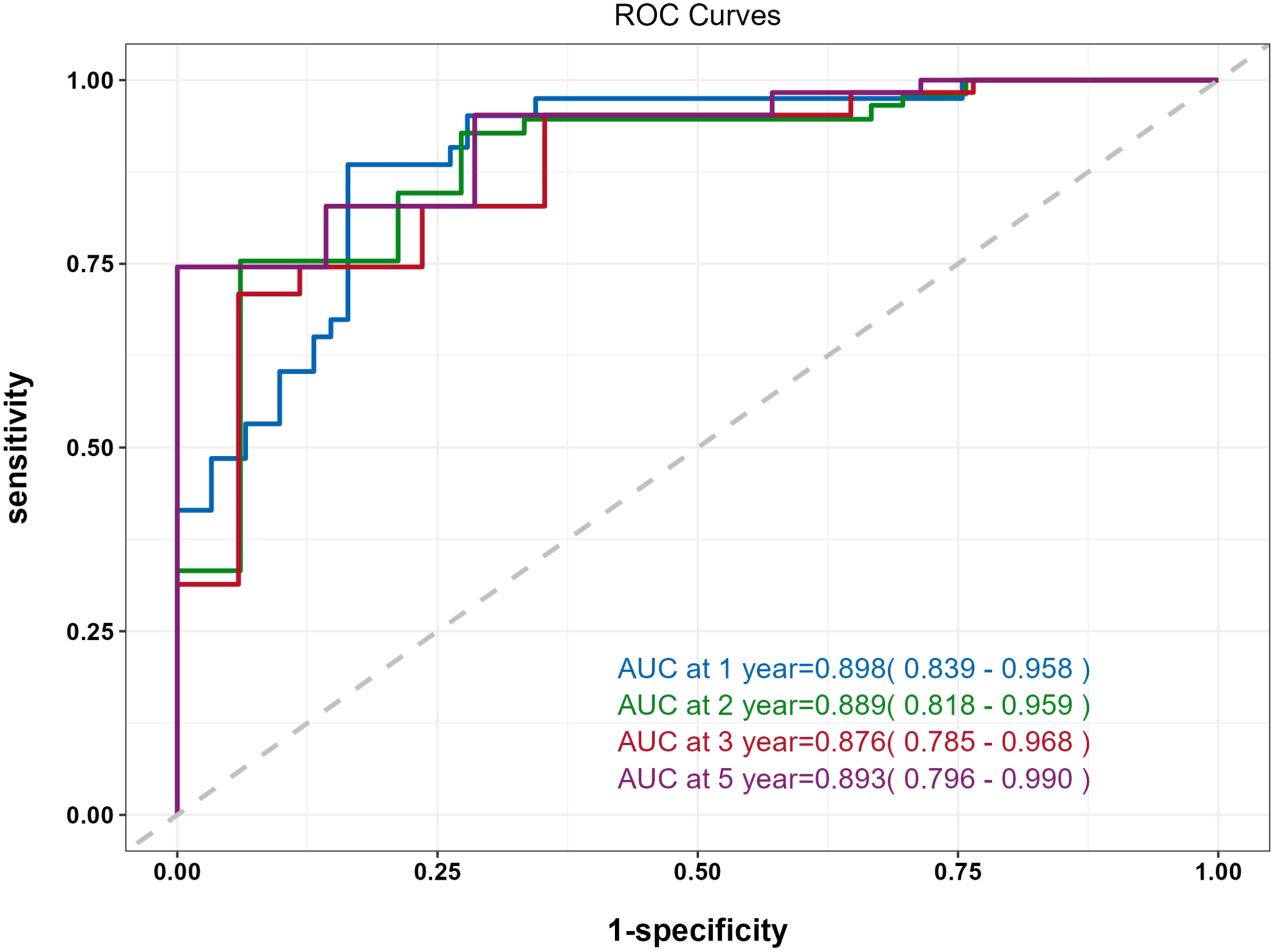
Time-dependent ROC (td-ROC) curve of the nomogram for predicting the progression to ESKD in DN patients.

**Figure 4:**
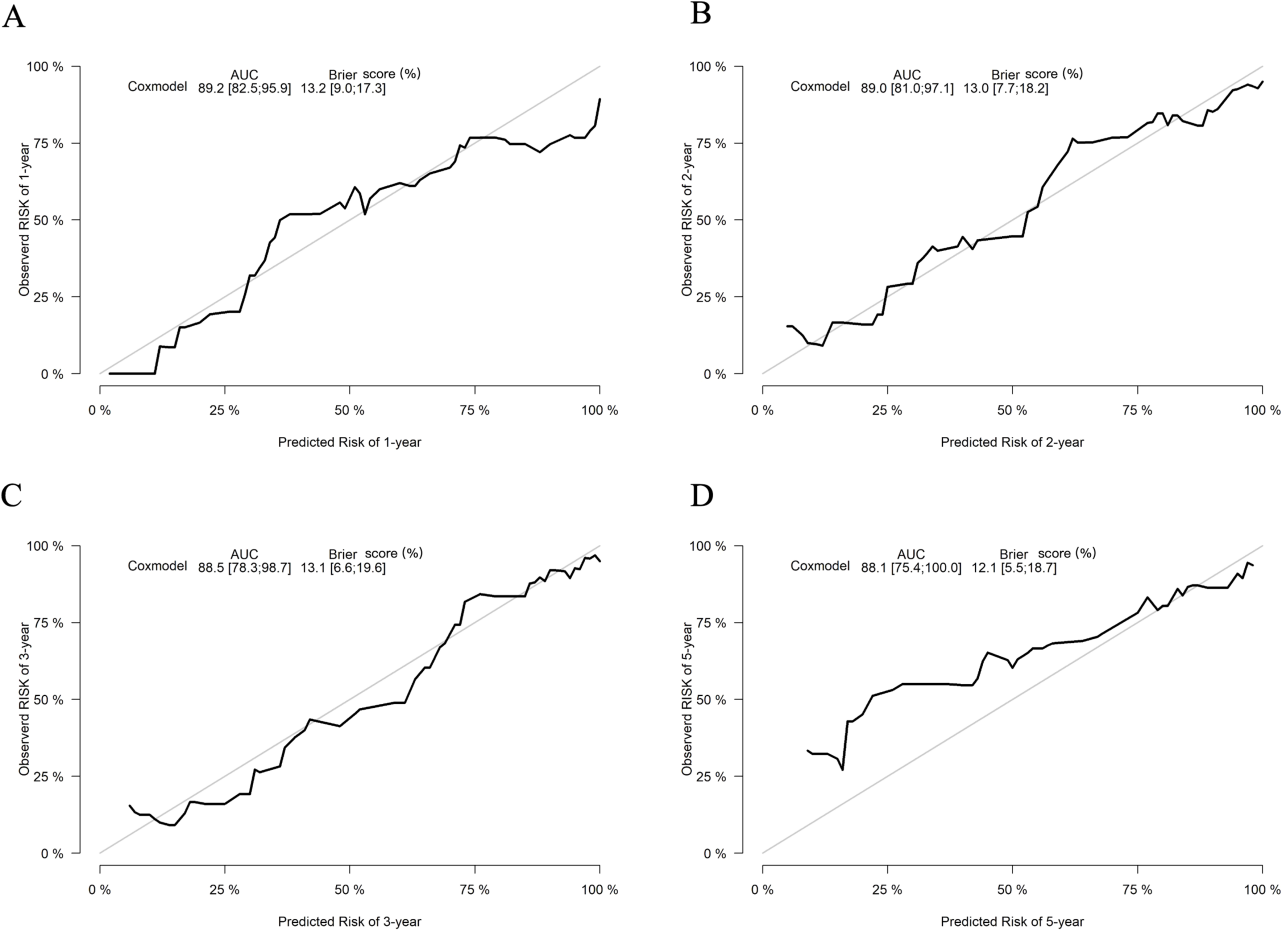
Calibration curves of the nomogram for predicting the progression to ESKD in DN patients. The calibration curves illustrate outcomes at 1 year **(A)**, 2 years **(B)**, 3 years **(C)**, and 5 years **(D)**. The x-axis represents the predicted odds of renal survival based on the nomogram, while the y-axis displays the observed actual renal survival.

**Figure 5:**
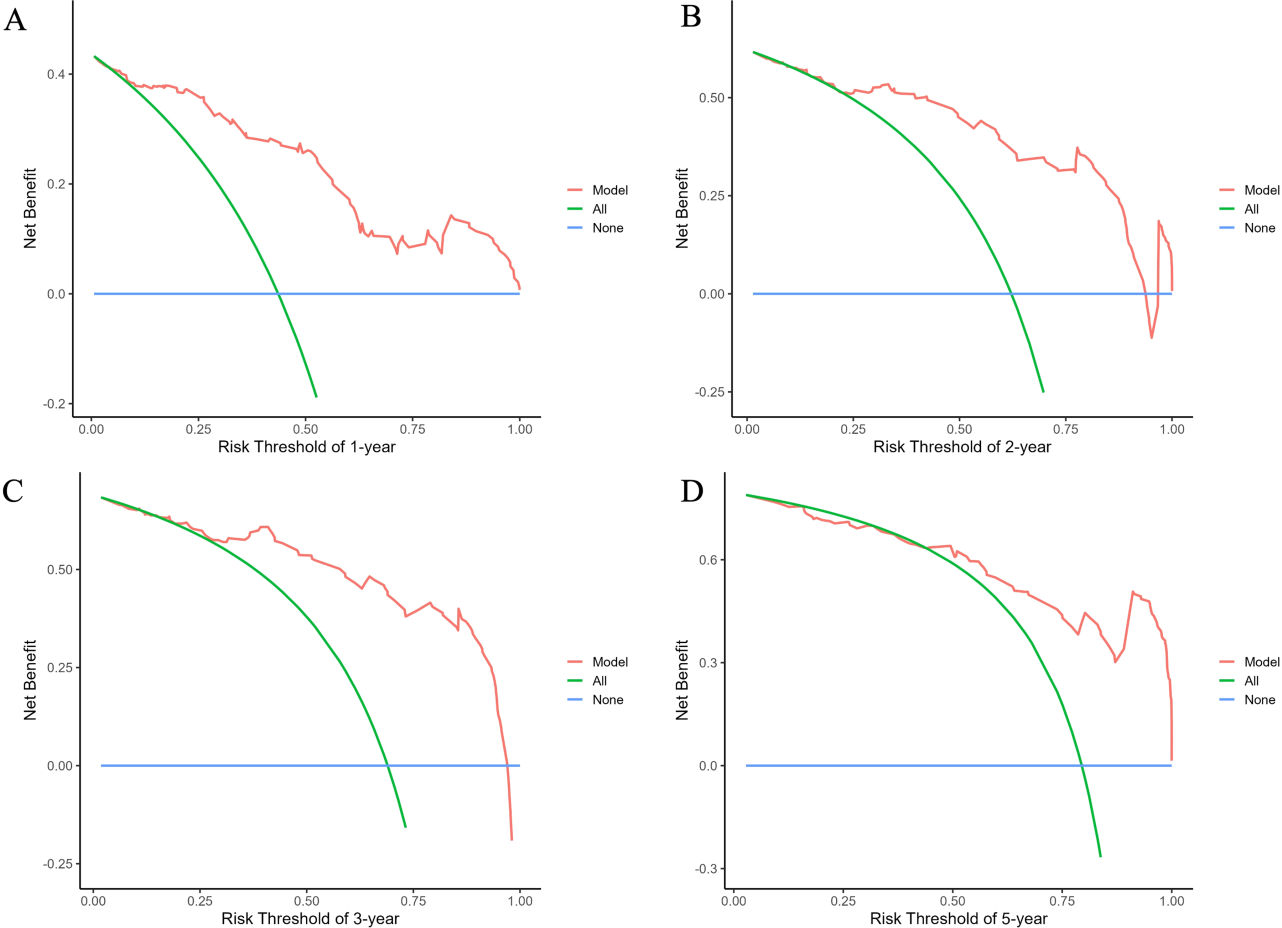
Decision curve analysis (DCA) curves of the nomogram for predicting the progression to ESKD in DN patients. The curves illustrate outcomes at 1 year **(A)**, 2 years **(B)**, 3 years **(C)**, and 5 years **(D)**. The net benefit curves for the nomogram are presented, with blue lines representing the net benefit when all DN patients are assumed not to progress to renal outcomes, and green lines indicating the net benefit when all DN patients are assumed to progress. The optimal model is defined as the one that provides the greatest net benefit at a given threshold.

The predictive performance of the nomogram was evaluated using the C-index, td-ROC curves, calibration curves, and DCA. The C-index of the nomogram was calculated to be 0.831 (95% CI: 0.787–0.876), indicating good predictive value. Next, the td-ROC curves were used to assess the model’s predictive performance at different time points. The AUC values for predicting the risk of ESKD at 1, 2, 3, and 5 years were 0.898 (95% CI: 0.839–0.958), 0.889 (95% CI: 0.818–0.959), 0.876 (95% CI: 0.785–0.968), and 0.893 (95% CI: 0.796–0.990), respectively (**Figure 3**), demonstrating strong predictive accuracy over time. The calibration curves for different time points showed that the predicted outcomes closely aligned with the actual outcomes (**Figure 4**). **Figure 5** illustrates the DCA curves, evaluating the net clinical benefit of the nomogram’s predictions at each time point. The results showed significant net benefit for clinical applications, with the red line positioned above both the “ALL” and “None” lines, indicating a favorable net benefit from interventions and supporting better clinical decision-making.

## 4 Discussion

As one of the leading causes of CKD worldwide, DN requires requires prompt and accurate identification of patients at risk for disease progression, which is critical for effective clinical decision-making. In this study, we developed a non-invasive, user-friendly prognostic model for DN, providing a practical and effective tool for the early identification of patients at risk for ESKD. Our analysis identified eGFR, lgACR, CysC, Hb, and Fib as independent risk factors in the prognosis of biopsy-confirmed DN. Using these variables, we developed a novel prognostic nomogram to accurately and quantitatively assess the risk of adverse renal outcomes at 1, 2, 3, and 5 years in individual patients. This baseline-data-based model offers a reliable scoring system that supplements clinical practice by assisting clinicians in early identification of high-risk individuals for ESKD progression.

Many factors are associated with the progression of CKD in patients with diabetes. The 2022 joint guidelines by the American Diabetes Association and Kidney Disease: Improving Global Outcomes highlight that decreasing eGFR and increasing albuminuria are major risk factors for CKD progression (12). In patients with DN, eGFR and albuminuria are clinically recognized as the most two important risk factors for renal function decline, given the limited subtypes of DN (13). Although certain pathological parameters, such as IFTA and mesangiolysis/microaneurysm, are considered to be associated with rapid renal function decline, their accuracy often depends on the expertise of the pathologist (7, 10). In addition to albuminuria, Li et al. have suggested that urinary renal tubular epithelial cells and casts, observed through manual urine sediment analysis, are also risk factors for poor prognosis in DN (14). However, due to the need for specialized personnel, this test is not commonly performed in many institutions. Furthermore, some studies suggested that not only baseline albuminuria levels but also changes in albuminuria are critically important. In large population studies, a doubling of the ACR within two years is associated with a 50%-100% increased risk of progression to renal failure (15, 16). A 21-year-long study has also shown that intensive, multifactorial, and target-driven therapies could slow the progression of DN to ESKD by reducing proteinuria (17). In the present study, we also found ACR and eGFR are independent risk factors of renal survival in DN patients, consistent with previous reports (10, 14).

Our study also identified CysC as a significant predictive parameter. Previous large-scale global studies assessing the risk of renal function decline in CKD patients have seldom included CysC, primarily due to its limited routine availability in laboratories worldwide (18, 19). It has been proposed that CysC might be particularly effective for detecting early nephropathy. Research has shown that some patients with microalbuminuria exhibit elevated CysC levels even when their GFR remains within the normal range (18, 20). CysC is superior to creatinine, as it is more sensitive to minor GFR reductions, increases even with normal creatinine levels, and does not require urine collection. This makes it valuable for the early detection and treatment of CKD. Several studies have demonstrated that CysC is sufficiently effective in clinical practice as a biomarker for detecting early kidney injury and distinguishing the severity of renal impairment in DN (21). Moreover, after adjusting for creatinine clearance, serum CysC levels are associated with factors such as higher HbA1c, older age, smoking, chronic inflammation, obesity, hypertension, and thyroid dysfunction, suggesting that CysC may also reflect these factors (22, 23). These factors may collectively influence the progression of renal function in DN patients. However, despite its usefulness, the routine clinical use of CysC is limited by its relatively high cost compared to traditional biomarkers such as serum creatinine. The measurement of CysC can be expensive, and its availability may be restricted in some healthcare settings. As a result, its widespread implementation in clinical practice is still challenging, particularly outside of specialized centers or clinical studies. The increased cost of using CysC may be a limiting factor for some institutions or healthcare systems, particularly in regions where budget constraints exist. Additionally, some institutions may not have the necessary infrastructure or equipment for reliable measurement, which further limits its widespread adoption. Nevertheless, given its potential to provide more accurate assessments of kidney function, its use could be considered in specific clinical contexts where precision is crucial. It’s surprising that age was not included in this model. This could be explained as small sample size of our cohort. Besides, chronological age does not always reflect clinically relevant biological age. Compared to chronological age, physiological age has a much stronger correlation with disability and mortality (24).

In our cohort, Hb and Fib levels are closely linked to renal function deterioration and showed good discrimination of DN patients at risk of progressing to ESKD. Anemia, commonly observed in CKD patients, often results from decreased erythropoietin production due to renal impairment (25). It has been reported as a significant risk factor for the progression of CKD, with studies indicating that its presence can accelerate the decline in renal function. Observational studies further suggested that in patients with diabetes and CKD, low Hb levels may increase the risk of kidney disease progression, cardiovascular events, and mortality, potentially due to mechanisms like underlying heart failure and renal ischemia resulted from reduced oxygen delivery (26, 27). Moreover, Zhao et al. found that DN patients with a rapid decline in eGFR had significantly lower Hb levels compared to those without rapid decline, identifying Hb as an important prognostic factor for rapid renal function decline in DN patients (7). These findings, consistent with our own, suggest the importance of monitoring and managing Hb levels to improve patient outcomes and slow CKD progression. Growing evidence indicates that Fib, a key acute-phase reactant involved in coagulation and inflammation, is elevated in various chronic diseases, including DN. Wang et al. observed that urinary Fib could predict the risk of CKD progression to ESKD, including in patients with CKD and diabetes (28). Cross-sectional studies in patients with type 2 diabetes have found that serum Fib levels are associated with decreased eGFR, proteinuria, and increased glomerular basement membrane thickness (29, 30). Subsequent study confirmed that serum Fib is an independent risk factor for ESKD in biopsy-proven DN patients (31). Consistent with previous reports, our findings show that elevated Fib levels in our cohort are significantly associated with a higher risk of progression to ESKD, reinforcing its role as a crucial prognostic marker in DN. Monitoring Fib levels may play a role in predicting and managing the progression DN, but further research is needed to better evaluate the benefits and risks of therapies aimed at reducing Fib levels.

It was surprising that poor glycemic control, which has been reported in the literature to be closely related to serum HbA1c levels and the progression to ESKD and all-cause mortality, was not found to be a significant risk factor in our study. This finding is consistent with previous reports (10, 32, 33). One possible explanation for the lack of difference in our study is that it was cross-sectional, and we did not follow up with patients. Therefore, the HbA1c values measured at the time of renal biopsy only reflect the patient’s glycemic control at that point. It is possible that patients who did not progress to ESKD may have better managed their blood sugar levels after the diagnosis of DN. Additionally, both of the hospitals included in this study are tertiary referral centers, where patients often present with more advanced and complex conditions, which can contribute to poorer glycemic control. Another reason for the lack of association is that many of our patients had already experienced significant kidney damage (with a median eGFR of 48.78 mL/min/1.73 m²). At this advanced stage of diabetic nephropathy, the influence of glycemic control on further kidney function decline may be diminished.

Nomograms, as a practical and visual predictive tool, are widely used in the prediction of CKD progression. Wu et al. collected clinical data and peripheral blood samples from 300 CKD stage 1 patients and identified hypertension, diabetes, and urinary albumin as significant factors in CKD progression' to improve clarity and conciseness (34). In another retrospective study, Zhang et al. included 309 CKD patients and found that hypoalbuminemia, proteinuria, elevated LDL, diabetes, hypertension, and CKD stage were risk factors for the progression to ESKD (35). Liao et al. included 416 diagnosed CKD patients and measured body composition areas through abdominal CT imaging, revealing that urea, diabetes, 24-hour urinary protein, mean arterial pressure, and subcutaneous adipose tissue radiodensity were valuable indicators for predicting CKD progression (36). These studies also developed nomograms. These studies, unlike ours, included CKD patients from all causes and identified diabetes as an independent risk factor for CKD progression. Our study, however, specifically focuses on patients with CKD caused by diabetes.

Our study does have some limitations. First, the patients were drawn from only two centers, and the sample size was relatively small. This limitation stems from our decision to include only biopsy-confirmed DN patients to minimize confounding factors, which both reduced our sample size and introduced selection bias. Since not all diabetic patients with kidney disease undergo renal biopsy, this both limited our sample size and introduced selection bias, meaning our findings may not be generalizable to the broader population of type 2 diabetes patients. Secondly, the small sample size also precluded us from validating the model externally, a significant limitation that requires further research with larger cohorts to confirm the model’s robustness. Additionally, this study was retrospective, and all participants were Chinese, which could contribute to selection bias and necessitate larger cohort studies to validate the applicability of this model. Another limitation is the relatively short follow-up period, which may not be sufficient to capture the full progression of DN, as kidney disease typically progresses over a longer duration. A longer follow-up would provide a more accurate understanding of the long-term outcomes in DN patients. Lastly, we did not collect data on the patient treatments, which are important factors that could have impacted the prognosis and introduced potential confounding variables in our results.

In summary, our study introduces a novel prediction model that significantly enhances the timely identification of DN patients at risk for progression to ESKD. The model includes five routine laboratory tests: eGFR, lgACR, CysC, Hb, and Fib, and demonstrated high predictive performance and good calibration. These factors not only play crucial roles in monitoring and assessing disease progression but also inform early intervention strategies. Implementing this prediction model in clinical practice holds promise for improving patient management, particularly by enabling clinicians to develop more personalized treatment plans. Future research should further investigate the specific roles of these biomarkers in different subtypes of DN and evaluate whether targeting these factors can improve long-term outcomes, thereby enhancing the management of DN patients.

## Data Availability

The raw data supporting the conclusions of this article will be made available by the authors, without undue reservation.
