# Supplementary material for: Development of a nomogram for predicting renal survival in patients with biopsy-proven diabetic nephropathy
Source: Front Endocrinol (Lausanne). 2025 Mar 27;16:1532494. doi: 10.3389/fendo.2025.1532494 (PMC11982824; doi:10.3389/fendo.2025.1532494)
Supplement: Supplementary file 1 [file Table1.docx]

Supplementary Table 1. Pathological characteristics of patients with diabetic nephropathy

| Variables | Total patients (*n*=140) | Progression to ESKD | | P |
| --- | --- | --- | --- | --- |
|  |  | Yes (*n*=81) | No (*n*=59) |  |
| Glomerular class, *n* (%) |  |  |  | <0.001 |
| I | 5（3.57） | 0（0） | 5（8.47） |  |
| IIa | 11（7.86） | 2（2.47） | 9（15.25） |  |
| IIb | 18（12.86） | 8（9.88） | 10（16.95） |  |
| III | 98（70） | 65（80.25） | 33（55.93） |  |
| IV | 8（5.71） | 6（7.41） | 2（3.39） |  |
| IFTA, *n* (%) |  |  |  | 0.001 |
| 0 | 1（0.71） | 0（0） | 1（1.69） |  |
| 1 | 37（26.43） | 15（18.52） | 22（37.29） |  |
| 2 | 39（27.86） | 20（24.69） | 19（32.2） |  |
| 3 | 63（45） | 46（56.79） | 17（28.81） |  |
